# Higher Prevalence of “Low T3 Syndrome” in Patients With Chronic Fatigue Syndrome: A Case–Control Study

**DOI:** 10.3389/fendo.2018.00097

**Published:** 2018-03-20

**Authors:** Begoña Ruiz-Núñez, Rabab Tarasse, Emar F. Vogelaar, D. A. Janneke Dijck-Brouwer, Frits A. J. Muskiet

**Affiliations:** ^1^Department of Laboratory Medicine, University Medical Centre Groningen, University of Groningen, Groningen, Netherlands; ^2^Healthy Institute, Madrid, Spain; ^3^European Laboratory of Nutrients, Bunnik, Netherlands

**Keywords:** chronic fatigue syndrome, thyroid, “low T3 syndrome”, triiodothyronine, reverse triiodothyronine, urinary iodine, inflammation, high-sensitive C-reactive protein

## Abstract

Chronic fatigue syndrome (CFS) is a heterogeneous disease with unknown cause(s). CFS symptoms resemble a hypothyroid state, possibly secondary to chronic (low-grade) (metabolic) inflammation. We studied 98 CFS patients (21–69 years, 21 males) and 99 age- and sex-matched controls (19–65 years, 23 males). We measured parameters of thyroid function, (metabolic) inflammation, gut wall integrity and nutrients influencing thyroid function and/or inflammation. Most remarkably, CFS patients exhibited similar thyrotropin, but lower free triiodothyronine (FT3) (difference of medians 0.1%), total thyroxine (TT4) (11.9%), total triiodothyronine (TT3) (12.5%), %TT3 (4.7%), sum activity of deiodinases (14.4%), secretory capacity of the thyroid gland (14.9%), 24-h urinary iodine (27.6%), and higher % reverse T3 (rT3) (13.3%). FT3 below the reference range, consistent with the “low T3 syndrome,” was found in 16/98 CFS patients vs. 7/99 controls (OR 2.56; 95% confidence interval = 1.00–6.54). Most observations persisted in two sensitivity analyses with more stringent cutoff values for body mass index, high-sensitive C-reactive protein (hsCRP), and WBC. We found possible evidence of (chronic) low-grade metabolic inflammation (ferritin and HDL-C). FT3, TT3, TT4, and rT3 correlated positively with hsCRP in CFS patients and all subjects. TT3 and TT4 were positively related to hsCRP in controls. Low circulating T3 and the apparent shift from T3 to rT3 may reflect more severely depressed tissue T3 levels. The present findings might be in line with recent metabolomic studies pointing at a hypometabolic state. They resemble a mild form of “non-thyroidal illness syndrome” and “low T3 syndrome” experienced by a subgroup of hypothyroid patients receiving T4 monotherapy. Our study needs confirmation and extension by others. If confirmed, trials with, e.g., T3 and iodide supplements might be indicated.

## Introduction

Chronic fatigue syndrome (CFS), also referred to as myalgic encephalomyelitis, is a complex heterogeneous disease, most commonly characterized by disabling fatigue, cognitive impairment, disrupted sleep and concomitant skeletal and muscular pain, lasting for more than 6 months and not improving with rest (1, 2) [for a broader definition, see Ref. (3)]. Impaired physical and social functioning, vitality, emotional well-being and role limitations due to emotional problems (4) contribute to an impaired quality of life (5). Although most patients have mild or moderate symptoms, some suffer from severe CFS and are housebound or even unable to move from their beds (4). The diagnosis of CFS is based on the Fukuda criteria, i.e., symptoms, disability, and exclusion of explanatory illnesses, and not by means of physical signs or abnormalities in laboratory test results (1–3). About 75% or more are female. The mean age of onset is 29–35 years and the mean illness duration ranges from 3 to 9 years (6), which implies that the symptoms are reversible. A meta-analysis of clinically confirmed cases in several countries indicates a prevalence of 0.76% (7). In 2005, the prevalence of CFS in The Netherlands was slightly lower, 0.18–0.25% (30,000–40,000 patients among 16 million inhabitants) (8).

The underlying cause of CFS remains unclear. Many pathophysiological cascades have been hypothesized but underlying organic conditions are rarely found. Disturbed hypothalamus–pituitary–adrenal (HPA) axis, presented as mild hypocortisolism, heightened negative feedback and blunted responses to challenge have been found in CFS (9). Computational analysis using endocrine and gene expression data suggest that CFS is associated with immune-mediated loss of thyroid function, exacerbated by a blunted HPA axis response (10). Autonomic dysfunction, including orthostatic intolerance and syncope, microglial activation and structural changes, indicate involvement of the brain (11). There is accumulating evidence that the cardiovascular system is compromised, with reports of autonomic dysfunction, attenuated heart rate and blood pressure (12) and increased death rate from heart failure (13). The latter finding was related to a blunted cortisol response (14). Taken together, dysfunctional central housekeeping involving interactions between both the HPA and hypothalamus–pituitary–thyroid (HPT) axes and the sympathetic/adrenal medulla, rather than single-hormone-axis disturbances, might play a key role in the development of CFS symptoms (10, 11, 14).

Dysregulation of the immune system in CFS may include autoimmune reactions and low-grade inflammation. Some studies demonstrated autoantibodies directed at diverse nuclear and neuronal components (15, 16) and against some neurotransmitters and neurotransmitter receptors in the CNS (17, 18). Others associated infection and vaccination with later CFS onset (19, 20). Recently, pandemic influenza A (H1N1) infection was related with a more than two-fold increased CFS risk (21). A state of low-grade inflammation (22), as derived from elevated (hs)CRP (23), interleukin (IL)-6 (24), IL-1 and tumor necrosis factor (TNF)-α (22), and/or nuclear factor kappa B (NFκB) (25) has, however, not consistently been found (26–28), possibly because of differences in experimental approaches and patient conditions (28). Increased translocation of lipopolysaccharides (LPS) from Gram-negative enterobacteria with subsequent gut-derived inflammation was also found (29). Giloteaux et al. demonstrated intestinal dysbiosis resulting from a more proinflammatory gut microbiome that may trigger the immune system (30). Recently, the relationship between the thyroid with gut microbiome and inflammation became apparent from the associations of both hypothyroidism and levothyroxine use with small intestinal bacterial overgrowth (31).

Several symptoms resemble those of hypothyroidism. They are, however, not accompanied by the marked thyrotropin (TSH) increases of full-blown hypothyroidism (32). Fuite et al. (10) suggested immune-mediated loss of thyroid function in CFS patients. Low-grade inflammation and subclinical hypothyroidism are not mutually exclusive. Inflammation virtually affects all hormonal axes (33), including the HPT axis (34). Profound changes in this axis occur in the “non-thyroidal illness syndrome (NTIS),” also referred to as “euthyroid sick syndrome,” which has notably been investigated in critically ill patients (35). As part of the acute phase response, this condition may reflect an adaptation to counteract excessive catabolism during severe illness (34). The most important clinical chemical features of mild to moderate NTIS are normal/low-normal TSH, low total triiodothyronine (TT3) and free T3 (FT3) levels, normal/high-normal total thyroxine (TT4), decreased peripheral conversion of T4 to T3, and increased reverse T3 (rT3) levels (36). Chronic inflammation in rodents increases the expression of deiodinase 3 (D3), which inactivates both T3 and T4 with concomitant production of 3,3′-diiodothyronine (T2) and rT3, respectively (34). A recent study (37) also reported elevated concentrations of 3,5-T2 in humans affected by cardiac NTIS.

Chronic fatigue syndrome has been described as an “allostatic overload condition” (38), where the physiological mechanisms employed to deal with stress (also named “allostatic states”) contribute to the perpetuation of the disorder. CFS patients are 1.9 times more likely to have a high allostatic load index than healthy controls (39) and this allostatic load also correlates positively with CFS symptoms (40). Thyroid allostasis-adaptive responses, presenting as NTIS, have been found in many conditions, ranging from critical illness, uremia and starvation to tumors (41). Taken together, it is possible that, despite TSH and T4 levels within reference ranges, CFS symptoms may be attributable in part to allostatic responses, i.e., lower thyroid hormone activity, secondary to chronic (low-grade) inflammation caused by, e.g., a compromised gut microbiome and gut wall integrity.

In the present case–control study, we focused on signs of low-grade inflammation and subclinical hypothyroidism. We measured parameters of thyroid function, low-grade inflammation and gut wall integrity (42), together with secondary markers of inflammation, also named metabolic inflammation (43, 44), including insulin resistance-mediated *de novo* lipogenesis (DNL), HDL-cholesterol (HDL-C), and the status of nutrients influencing thyroid function (iodine and selenium) and inflammation [fish oil fatty acids (FA) and vitamin D].

## Materials and Methods

### Study Design and Study Group

**Figure 1:**
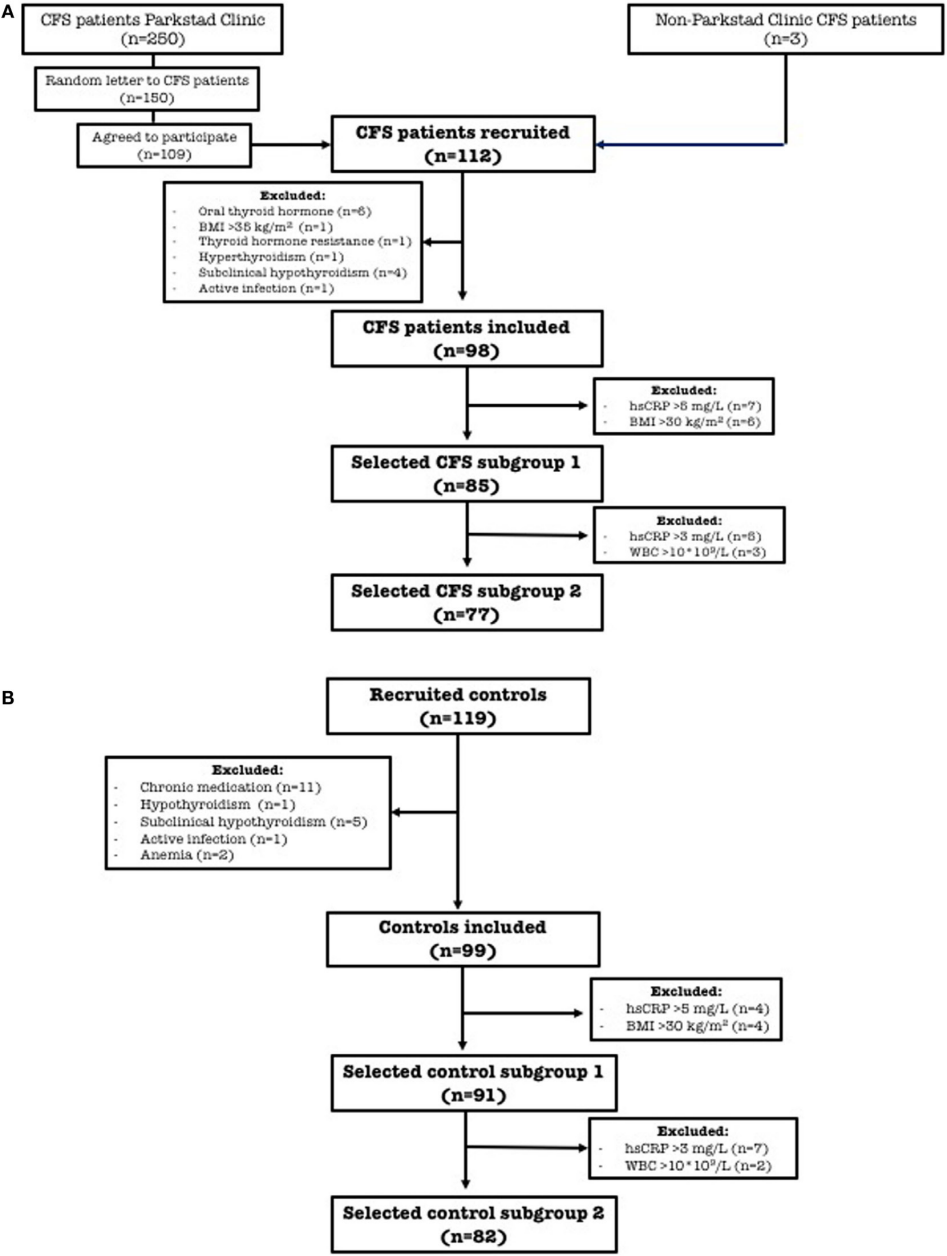
Flow-chart: inclusion of chronic fatigue syndrome patients **(A)** and controls **(B)** in the different groups and subgroups. Abbreviations: CFS, chronic fatigue syndrome; *n*, number of subjects; BMI, body mass index; hsCRP, high-sensitive C-reactive protein; WBC, white blood cells.

Patients were recruited in the Parkstad Clinic in Amsterdam, The Netherlands. They were diagnosed with CFS according to the CBO guideline (45). These are based on the Fukuda criteria (1), with the exclusion criteria of Reeves (3). In the Parkstad Clinic, 250 CFS patients are seen on a regular basis. From these, 150 were randomly selected to receive a letter requesting their voluntary participation. A total of 109 agreed to participate. Three of the participants were not patients of the Parkstad Clinic, making a total of 112 (see Figure 1 for flow scheme). The patients completed a questionnaire on their health, recent non-chronic medication use, smoking habits, supplement use, and pregnancy and lactation. Exclusion criteria were use of medication that may affect thyroid function (e.g., T4, antiarrhythmic drugs, such as amiodarone or corticosteroids), pregnancy, breastfeeding, and menstruation during urine collection. Other exclusion criteria were (biochemical) abnormalities that are excluded according to the CBO guideline and not demonstrated at the time of diagnosis, e.g., severe obesity [body mass index (BMI) > 35 kg/m^2^], infection [high-sensitive C-reactive protein (hsCRP) > 10 mg/L and white blood cells (WBC) > 10 × 10^9^/L], anemia [hemoglobin (Hb) < 7.0 mmol/L in women and < 8.0 mmol/L in men], hyperthyroidism [TSH below reference range with FT3 and/or free thyroxine (FT4) above reference range (46)], thyroid hormone resistance [elevated FT4 with non-suppressed TSH (47)], hypothyroidism (TSH above upper limit of reference range with FT4 below reference range), and subclinical hypothyroidism [TSH above reference range with normal FT4 (46)]. Weights and lengths were measured on the spot. Data on age were obtained from interviews in the Dutch language.

A total of 119 age- and sex-matched apparently healthy controls were recruited by advertisement in the city of Groningen, The Netherlands. Health was self-reported with the aid of a health checklist filled out before inclusion. Primary exclusion criteria were the use of any chronic medication, menstruation during urine collection, severe obesity (BMI > 35 kg/m^2^), and both pregnancy and breastfeeding. Incidental use of analgesics and short-term medication (e.g., antibiotics, more than 4 weeks ago) were allowed. Secondary exclusion criteria were infection (hsCRP > 10 mg/L and WBC > 10 × 10^9^/L), anemia (Hb < 7.0 mmol/L in women and < 8.0 mmol/L in men), hyperthyroidism [TSH below reference range with FT3 and/or FT4 above reference range (46)], thyroid hormone resistance [elevated FT4 with non-suppressed TSH (47)], hypothyroidism (TSH above upper limit of reference range with FT4 below reference range), and subclinical hypothyroidism [TSH above reference range with normal FT4 (46)]. Data on age were obtained from interviews in the Dutch language. Weight and height were self-reported.

All patients and controls received a verbal and written explanation of the objectives and procedures and all provided us with written informed consent. The study was in agreement with the ethical standards of the responsible committee on human experimentation (institutional and national) and with the Helsinki Declaration of 1975, as revised in 2008. The protocol was approved by the University Medical Center Groningen (UMCG) Medical Ethical Committee (NL44299.042.13, METc 2013/154, dated August 12, 2013).

### Sample Size and Final Study Groups

The calculation of the sample size (i.e., 100 subjects per group) was based on the correlation coefficient of a comparable population using different steps [for more information, see Ref. (48)]. For this, we used the correlation coefficient found by Girvent et al. (49) for the association of both inflammatory markers CRP and IL-6 with rT3, choosing the highest (i.e., *r* = 0.75 for rT3 vs. CRP). In this study, subjects with NTIS were compared with patients without euthyroid sick syndrome, both undergoing surgery. Assuming a 95% confidence interval (CI) of (0.59, 0.79), we estimated the sample size using IBM SPSS Statistics (version 20), with the obtained formula, where the *n* (sample size) appeared inside the Euler number exponent (e). We anticipated 20% exclusion based on abnormal laboratory data, and therefore aimed at the initial inclusion of 120 patients and controls.

We gathered information about supplement intake (vitamin D and fish oil) from 71/98 CFS patients. Users were defined as supplementing themselves either with multivitamins and/or other supplements containing that specific nutrient.

Subsequently, we performed a sensitivity analysis applying stricter exclusion criteria for possible signs of (low-grade) inflammation (“selected groups 1 and 2,” see Results and Figure 1). In the first sensitivity analysis, both CFS patients and controls with BMI > 30 kg/m^2^ and/or hsCRP > 5 mg/L were excluded. In the second one, we also excluded subjects with hsCRP > 3 mg/L and/or with WBC > 10 × 10^9^/L.

### Sample Collection and Analyses

Approximately 50 mL of blood were collected by venipuncture in the non-fasting state in three types of tubes (EDTA anticoagulated, lithium–heparin anticoagulated, and serum separator). Samples were processed within 2 h after collection. Twenty-four-hour urine samples were collected and their volumes measured. Samples were stored at −20°C and sent to the participating laboratories [UMCG, laboratory of Special Chemistry and Radiochemistry, Academic Medical Center in Amsterdam (AMC), Medical Laboratories, Reinier de Graaf Groep Diagnostisch Centrum, Delft, and European Laboratory of Nutrients (ELN), Bunnik].

EDTA-whole blood was used for the measurement of routine hematological parameters [Hb, hematocrit, WBC, red blood cells (RBC), and thrombocytes] with a Sysmex XN-9000 Hematology Analyzer (Sysmex Nederland BV, Etten Leur, The Netherlands). The remainder of the EDTA blood was separated into thrombocyte-rich plasma and an RBC pellet by centrifugation for 10 min at 1,800 *g*. RBC were washed three times with 0.9% NaCl and resuspended to an about 50% hematocrit. After washing, 200 µl of the RBC suspension was transferred to a teflon-sealable “Sovirel” tube containing 2 mL of methanol-6 mol/L HCl (5:1 v/v), 1 mg butylated hydroxytoluene (antioxidant), and 50 µg 17:0 (internal standard). In this ready-to-transmethylate mixture, FAs are stable at room temperature and in the dark for months (50). After centrifugation (10 min, 1,800 *g*) of the thrombocyte-rich EDTA-plasma, we aliquoted the isolated thrombocyte-poor EDTA plasma and stored it in 2 mL cryovials at −20°C. Lithium–heparin whole blood (1.5 mL) was aliquoted for measurement of elements. The remainders of the lithium–heparin anticoagulated blood and the coagulated blood sample were centrifuged for 10 min at 1,800 *g*. The resulting plasma and serum were isolated, transferred to 2 mL cryovials, and stored at −20°C until analysis.

Red blood cell–FA compositions were determined by capillary gas chromatography/flame ionization detection in the UMCG, using previously described procedures (50). RBC-FA contents were expressed in g/100 g FA (g%). Tryptophan and kynurenine were measured in EDTA-plasma by LC–electrospray ionization–MS/MS as previously described (51). Serum 25(OH)D2 and 25(OH)D3 [together referred to as 25(OH)D] were determined by isotope dilution-online solid-phase extraction liquid chromatography–tandem mass spectrometry (ID-XLC-MS/MS) in the UMCG (52). Plasma MMA was measured by LC-MS/MS according to Nelson et al. (53). Serum iron, ferritin, hsCRP, total cholesterol (TC), and LDL- and HDL-C were measured using a Roche Modular P module (Roche, Almere, The Netherlands). Vitamin B12, folate, TSH, FT4, and FT3 were assayed by electrochemiluminescent immunoassay on the Roche Modular E170 Analyzer. Serum TT4 and TT3 were measured using an Architect i2000SR (Abbott Diagnostics, Hoofddorp, The Netherlands). Serum antithyroglobulin antibodies and antithyroid peroxidase antibodies were measured with an Immulite 2000 (Siemens, The Netherlands). Plasma rT3 was measured by in-house RIA (54) at the AMC, The Netherlands. Plasma homocysteine was analyzed in the UMCG by competitive protein binding assays with the use of an immunochemistry analyzer (IMX; Abbott Diagnostics, Hoofddorp, The Netherlands).

Whole blood- and lithium–heparin plasma selenium, copper, magnesium and zinc and iodine in urine were measured using ICP-MS 7700x (Agilent, Amstelveen, The Netherlands) in the ELN. Selenium, copper, magnesium and zinc contents in RBC were calculated from their concentrations in plasma and whole blood, using hematocrit values for correction. Plasma zonulin (active form) concentrations were measured using the K5600 ELISA kit (Immundiagnostik AG, Bensheim, Germany). The quantification of 8-iso-prostaglandin F2-isoprostanes in urine was performed by GC-tandem-MS using a two-step derivatization and a selective solid-phase extraction protocol with HLB and Silica columns as described by Zhao et al. (55). The tryptophan/kynurenine ratio was calculated. This ratio may be decreased during inflammation (56, 57).

For the investigation of the pathogenesis of the “low-T3 syndrome,” we measured FT3/FT4, TT3/TT4 and rT3/TT3 ratios. For the investigation of the underlying etiology of the “low-T3 syndrome,” we calculated the following variables of thyroid metabolism: standard TSH index (sTSHi), in order to quantify the thyrotropic function of the pituitary (58); the sum activity of deiodinases [structure parameter inference approach (SPINA)-GD] as a variable for deiodination function (59); the secretory capacity of the thyroid gland (SPINA-GT), as an evaluation of thyroid secretory status (59); and the ratios of TT3/FT3 and TT4/FT4 as evaluations of protein binding of thyroid hormones. The sTSHi was calculated as TSHi = (TSH − 2.70)/0.676 (58). SPINA-GD and -GT were calculated as SPINA-GD = [β_31_ × (*K*_M1_ + FT4) × TT3]/(α_31_ × FT4) and SPINA-GT = [β_T_ × (*D*_T_ + TSH) × TT4]/(α_T_ × TSH). Constants in the equations were as follows: β_31_ = 8 × 10^–6^/s, *K*_M1_ = 5 × 10^–7^ mol/L, α_31_ = 0.026/L, β_T_ = 1.1 × 10^−6^/s, *D*_T_ = 2.75 mU/L, and α_T_ = 0.1/L (59, 60). The rT3/TT3 ratio was also calculated as a proxy for a metabolic shift. For the latter, we also calculated the %TT4, %TT3, and %rT3 by dividing their concentrations by the sum of TT4 + TT3 + rT3 and adjusting to 100%. Zinc/copper, TC/HDL-C and eicosapentaenoic acid (EPA)/arachidonic acid (AA) ratios were also calculated. A proxy for hepatic DNL (DNL liver) was calculated according to Kuipers et al. (61) (sum of RBC 16:0, 16:1ω7, 18:1ω7, 20:1ω7, 18:1ω9, 20:1ω9, and 22:1ω9). The omega-3 index, RBC-EPA + docosahexaenoic acid (DHA) (RBC-EPA + DHA) was calculated.

### Statistics

Statistical analyses were performed with IBM SPSS Statistics 23 SPSS Inc., Chicago, IL, USA. Mann–Whitney *U*-tests were used for the evaluation of between-group differences in the distribution. The Chi-square tests were used for the evaluation of between-group differences in nominal variables. Odds ratios were calculated to quantify the strength of the presence of low T3 in the different groups. Correlation analyses were performed using Spearman’s Rho for non-parametric variables.

## Results

Of the 112 initially included CFS patients, six taking oral thyroid hormone and one with BMI > 35 kg/m^2^ were excluded, leaving 105 patients. Of these, one subject with thyroid hormone resistance [defined as elevated serum levels of FT4 with non-suppressed TSH (47)], one with hyperthyroidism [TSH below reference range with FT3 and/or FT4 above reference range (46)], four with subclinical hypothyroidism [TSH above reference range with normal FT4 (46)], and one suspected of active infection (both hsCRP > 10 mg/L and WBC > 10 × 10^9^/L) were excluded; making a total of 98 finally included CFS patients (Figure 1).

Of the 119 age- and sex-matched apparently healthy controls, 11 taking chronic medication were excluded, leaving 108 controls. Of these, one with hypothyroidism (TSH above reference range with FT4 below reference range), five with subclinical hypothyroidism [TSH above reference range with normal FT4 (46)], one suspected of active infection (both hsCRP > 10 mg/L and WBC > 10 × 10^9^/L), and two with anemia were excluded; making a total of 99 finally included healthy controls (Figure 1).

### Whole Study Group

Characteristics of the 98 CFS patients and the 99 controls are shown in Table 1. The CFS patients (21 males, 77 females) had a median age of 43 years (range 21–69), median height of 172 cm (149–198), median weight of 68 kg (48–118), and median BMI of 22 kg/m^2^ (18–34). The age- and-sex-matched healthy controls (23 males, 76 females) had a median age of 39 years (19–65), median height of 173 cm (156–193), median weight of 70 kg (47–100), and a median BMI of 23 kg/m^2^ (18–33). The above anthropometric characteristics exhibited no between-group differences.

**Table 1:** Anthropometrics and laboratory data of 98 CFS patients and 99 controls.

		CFS patients	Controls			CFS patients		Controls	
	Units	Median (range)	Median (range)	*p*-Value	Reference range/cutoff value	% (*n*) below	% (*n*) above	% (*n*) below	% (*n*) above
**Anthropometrics**									
Number		98	99						
Gender	Male/female	21/77	23/76						
Age	Years	43 (21–69)	39 (19–65)	0.235					
Height	cm	172 (149–198)	173 (156–193)	0.996					
Weight	kg	68 (48–118)	70 (47–100)	0.618					
BMI	kg/m^2^	22 (18–34)	23 (18–33)	0.384	<30	–	9(9)	–	4 (4)

**Thyroid function**									
TSH	mU/L	1.43 (0.49–4.40)	1.59 (0.53–3.32)	0.527	0.5–4	1 (1)	1 (1)	0 (0)	0 (0)
FT4	pmol/L	15.9 (11.4–23.0)	15.6 (11.0–19.7)	0.562	11.0–19.5	0 (0)	5 (5)	0 (0)	1 (1)
FT3	pmol/L	5.2 (3.9–6.9)	5.2 (3.2–12.8)	0.047*	4.4–6.7	16 (16)	2 (2)	7 (7)	17 (17)
TT4	nmol/L	63.4 (17.8–121.3)	72.0 (45.4–134.8)	<0.001**					
TT3	nmol/L	1.4 (0.4–2.5)	1.6 (1.2–2.3)	<0.001**					
rT3	nmol/L	0.23 (0.08–0.40)	0.23 (0.12–0.41)	0.783	0.11–0.44	1 (1)	0 (0)	0 (0)	0 (0)
% TT4		97.55 (96.69–98.44)	97.55 (96.61–98.47)	0.513					
% TT3		2.04 (1.21–2.94)	2.14 (1.24–3.12)	0.012*					
% rT3		0.34 (0.12–1.14)	0.30 (0.15–0.45)	<0.001**					
TT3/TT4 ratio	mmol/mol	21.0 (12.3–30.4)	21.93 (12.62–32.26)	0.013*					
FT3/FT4 ratio	mol/mol	0.32 (0.20–0.49)	0.34 (0.24–0.74)	0.004**					
rT3/TT3 ratio	mol/mol	0.18 (0.05–0.60)	0.15 (0.08–0.24)	<0.001**					
TT3/FT3 ratio	mol/mmol	0.28 (0.08–0.42)	0.31 (0.13–0.45)	<0.001**					
TT4/FT4 ratio	mol/mmol	4.08 (1.26–6.84)	4.62 (3.15–9.05)	<0.001**					
SPINA-GT	pmol/s	1.77 (0.37–4.36)	2.08 (1.07–6.43)	0.010*					
SPINA-GD	nmol/s	13.42 (4.36–23.89)	15.67 (10.15–25.05)	<0.001**					
sTSHi		−1.89 (−3.27–2.51)	−1.65 (−3.21–0.92)	0.527					

**Inflammation**									
WBC	10^9^/L	6.1 (3.3–11.7)	6.3 (3.7–12.0)	0.182	4–10	5 (5)	5 (5)	3 (3)	5 (5)
hsCRP	mg/L	0.94 (0.09–8.28)	0.77 (0.11–21.62)	0.254	< 5.0	–	7 (7)	–	4 (4)
Kynurenine	μmol/L	1.63 (0.79–2.97)	1.81 (0.94–3.03)	0.001**	1.14–3.02	14 (14)	0 (0)	3 (3)	1 (1)
Tryptophan	μmol/L	54.0 (27.9–88.7)	56.4 (30.9–98.6)	0.003**	45–83	19 (19)	1 (1)	3 (3)	2 (2)
Tryptophan/kynurenine	mol/mol	32.57 (18.47–63.78)	32.42 (17.43–56.92)	0.443					
Ferritin^a^	μg/L	77 (8–600)	52 (5–386)	0.007*	Men 30–400	1 (1)	1 (1)	0 (0)	0 (0)
Urinary isoprostanes	nmol/d	1,271 (164–6,830)	1,336 (170–9,978)	0.373	Women 15–130	1 (1)	10 (10)	12 (12)	13 (13)
TC	mmol/L	5.2 (2.8–7.6)	5.1 (3.0–9.1)	0.627					
HDL-C^b^	mmol/L	1.4 (0.6–3.9)	1.6 (0.7–3.2)	<0.001**					
LDL-C	mmol/L	3.1 (1.1–5.6)	3.1 (1.1–7.0)	0.792					
TC/HDL-C^b^	mol/mol	3.5 (1.7–10.7)	3.1 (1.7–9.0)	0.001**					
DNL liver	g%	34.98 (32.37–43.29)	36.26 (33.58–43.85)	<0.001**					

**Intestinal permeability**									
Zonulin	ng/mL	1.24 (0.17–2.27)	1.39 (0.25–2.89)	0.002**					

**Nutritional factors**									
Urinary iodine (24 h)	μg/d	113 (20–559)	156 (27–666)	<0.001**	>200	87 (85)	11 (11)	66 (65)	22 (22)
Selenium (P)	mg/L	0.08 (0.05–0.27)	0.09 (0.06–0.46)	0.103	0.08–0.30	42 (42)	0 (0)	32 (32)	1 (1)
Selenium (IC)	mg/L	0.17 (0.11–0.97)	0.15 (0.04–0.31)	0.001**	0.17–0.55	45 (44)	2 (2)	62 (61)	0 (0)
25 (OH) vitamin D	nmol/L	75.8 (16.0–217.2)	54.9 (5.4–133.4)	<0.001**	80–250	59 (58)	0 (0)	83 (82)	0 (0)
RBC-EPA + DHA	g%	4.08 (1.95–7.81)	4.07 (1.91–8.54)	0.884	>8	100 (99)	0 (0)	98 (97)	2 (2)
RBC EPA/AA	g%	0.04 (0.01–1.00)	0.04 (0.01–0.18)	0.288					

*Data are medians (ranges). Mann–Whitney *U*-tests were used for between-group differences in the distribution*.

*^a^When analyzed according to gender, ferritin was higher in both male and female CFS patients as compared to controls (data not shown)*.

*^b^When the TC/HDL-C ratio and HDL-C were evaluated according to gender, we did not find differences in the TC/HDL-C ratio in the relatively small number of men, but those in the females persisted. HDL-C was lower in both male and female CFS patients compared to controls (data not shown)*.

*Significant at p < 0.05.

**Significant at p < 0.01.

*WBC, white blood cells; RBC, red blood cells; hsCRP, high-sensitive C-reactive protein; P, plasma; IC, intracellular; TSH, thyrotropin; FT4, free thyroxin; FT3, free triiodothyronine; TT4, total thyroxin; TT3, total triiodothyronine; rT3, reverse T3; GD, sum activity of deiodinases; GT, secretory capacity of the thyroid gland; sTSHi, standard TSH index; TC, total cholesterol; LDL-C, low-density lipoprotein-cholesterol; HDL-C, high-density lipoprotein-cholesterol; AA, arachidonic acid; EPA, eicosapentaenoic acid; DHA, docosahexaenoic acid; DNL, *de novo lipogenesis*, sum of RBC 16:0,16:1ω7, 18:1ω7, 20:1ω7, 18:1ω9, 20:1ω9 and 22:1ω9, according to Ref. (61)*.

#### Thyroid Hormones

Chronic fatigue syndrome patients exhibited lower FT3, TT4, TT3, %TT3, SPINA-GD, and SPINA-GT, lower ratios of TT3/TT4, FT3/FT4, TT3/FT3, and TT4/FT4; and higher %rT3 and rT3/TT3 ratio. There were no between-group differences in other thyroid hormone parameters, notably TSH, FT4, rT3, and %TT4 (Table 1). FT3 below the reference range was more frequently found in CFS patients (16/98) as compared to controls (7/99; *p* = 0.035) with an odds ratio of 2.56 (95% CI = 1.00–6.54).

#### (Metabolic) Inflammation

We did not find significant differences in WBC, hsCRP, tryptophan/kynurenine ratio and urinary isoprostanes. CFS patients displayed lower kynurenine and tryptophan, as compared to the healthy controls. Taking both genders together, we found that ferritin was higher in CFS patients as compared to controls. Analyzed according to gender, we found that ferritin was higher in both male and female CFS patients, as compared to their apparently healthy counterparts (females: 77 CFS vs. 76 controls; *p* = 0.003; males 21 CFS patients vs. 23 controls; *p* = 0.012) (data not shown in Table 1). Taking both genders together, we found that HDL-C was lower and the TC/HDL-C ratio higher in CFS patients as compared to controls. Analyzed according to gender, we found that HDL-C was lower in both male and female CFS patients, as compared to their apparently healthy counterparts (females: 77 CFS vs. 76 controls; *p* < 0.001; males 21 CFS patients vs. 23 controls; *p* = 0.04). The TC/HDL-C ratio was higher in female CFS patients compared to controls (*p* = 0.001) data not shown in Table 1. The RBC-FA composition showed a lower hepatic DNL in CFS patients.

Zonulin, a parameter of intestinal permeability (42), was lower in CFS patients as compared to controls.

#### Nutritional Factors Influencing Thyroid Function and Inflammation

The 24-h urinary iodine output, as a proxy of iodine status, was lower in CFS patients. Plasma selenium was similar, but intracellular selenium was higher (Table 1).

Vitamin D [25(OH)D] status of CFS patients was higher. Nevertheless, 58 patients (59%) and 82 (83%) controls presented 25(OH)D levels below the optimal cutoff of 80 nmol/L. None of the patients and 2% of the controls exhibited RBC-EPA + DHA contents above 8 g%, which is considered to confer optimal protection against cardiovascular (62) and neuropsychiatric (63) diseases. CFS patients and controls exhibited no differences in RBC-EPA/AA ratio, which is a risk factor for cardiovascular disease (64) and inflammation-induced depression (65). We gathered information about supplement intake from 71/98 CFS patients. Among these, the users did not exhibit higher status of vitamin D [plasma 25(OH)D; 30 vs. 41; *p* = 0.48] or EPA + DHA (RBC-EPA + DHA; 7 vs. 64; *p* = 0.44).

Additional laboratory data, including hematological indices, nutrient status influencing anemia, and other RBC-FA can be found in Table S1 in Supplementary Material.

#### Interim Conclusions Based on the Whole Group

**Figure 2:**
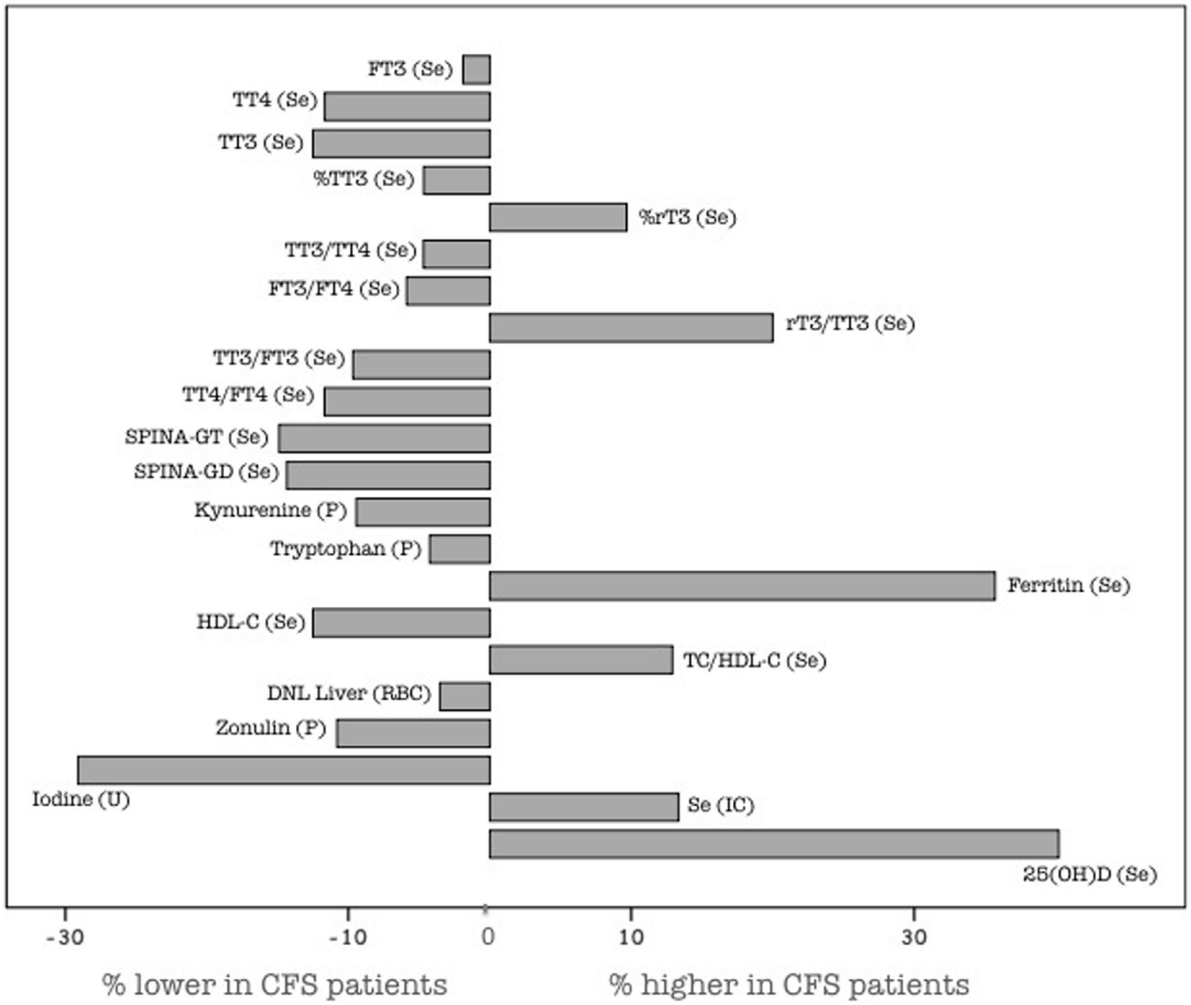
Between-group differences in parameters, depicted as percentages relative to control. Only parameters exhibiting significant between-group differences are depicted (see Table 1). Data are calculated from the medians (Table 1) according to [(median CFS − median controls)/median controls × 100] (in %). Se, serum; P, plasma; IC, intracellular; U, urinary; FT4, free thyroxin; FT3, free T3; TT4, total T4; TT3, total T3; rT3, reverse T3; TC, total cholesterol; HDL-C, high-density lipoprotein-cholesterol; DNL, *de novo* lipogenesis, sum of 16:0, 16:1ω7, 18:1ω7, 20:1ω7, 18:1ω9, 20:1ω9, and 22:1ω9, according to Ref. (61); SPINA-GD, sum activity of deiodinases; SPINA-GT, secretory capacity of the thyroid gland; sTSHi, standard TSH index.

Figure 2 shows the case–control between-group differences in terms of percentages lower or higher of the medians of CFS patients compared to those of controls. Only significant between-group differences are depicted. Most remarkably, the results for the whole group indicated similar TSH in patients with CFS, but subtle changes in thyroid hormone concentrations, with an apparent shift in their metabolism. CFS patients notably exhibited relatively lower FT3, TT4, and TT3; lower deiodination function (SPINA-GD), lower thyroid secretory function (SPINA-GT), lower protein binding of thyroid hormones (TT3/FT3, TT4/FT4), and lower T3/T4 hormone ratios (TT3/TT4, FT3/FT4), lower %TT3, higher %rT3, and higher rT3/TT3 ratio. The lower 24-h urine iodine output of CFS patients was also remarkable.

### Sensitivity Analyses

The strength of the above findings for the whole group was tested with “sensitivity analyses.” For this goal, we created “selected subgroups 1 and 2” by exclusion of subjects with the most prominent signs of (metabolic) inflammation, defined as relatively high hsCRP and BMI.

#### Selected Subgroups 1

Of the 98 CFS patients, seven with hsCRP > 5 mg/L (of whom three had BMI > 30 kg/m^2^) and six others with a BMI > 30 kg/m^2^ were excluded to create a subgroup of 85 patients (selected CFS subgroup 1) (Figure 1). Of the 99 healthy controls, four with hsCRP > 5 mg/L and four others with a BMI > 30 kg/m^2^ were excluded to create a subgroup of 91 controls (selected control subgroup 1) (Figure 1). All parameters of thyroid function and inflammation that were significantly different between CFS patients and controls in the whole group, remained significant after this sensitivity analysis.

#### Selected Subgroups 2

Of the 85 CFS patients in selected subgroup 1, five with hsCRP > 3 mg/L and three with WBC > 10 × 10^9^/L (as more sensitive markers of inflammation) were excluded to create a subgroup of 77 patients (selected CFS subgroup 2) (Figure 1). Of the 91 controls in selected subgroup 1, 7 subjects with hsCRP > 3 mg/L and 2 with WBC > 10 × 10^9^/L were excluded to create a subgroup of 82 controls (selected control subgroup 2) (Figure 1). Characteristics of these CFS patients and controls, together with their clinical chemical data are shown in Table S2 in Supplementary Material. FT3 below the reference range was more frequently found in CFS patients (16/77) as compared to controls (7/82; *p* = 0.024) with an odds ratio of 2.81 (95% CI = 1.09–7.27), although the FT3 was no longer significantly lower. The higher ferritin proved no longer significantly different (compared to sensitivity analysis 1). However, ferritin remained higher in male and female CFS patients as compared to their apparently healthy counterparts (females: 59 CFS vs. 60 controls; *p* = 0.015; males 18 CFS patients vs. 22 controls; *p* = 0.026) (data not shown in Table S2 in Supplementary Material). Analyzed according to gender, we found that HDL-C remained lower and the TC/HDL-C ratio remained higher in female CFS patients as compared to the healthy controls (females: 59 CFS vs. 60 controls; *p* = 0.010 and *p* = 0.007) (data not shown in Table S2 in Supplementary Material).

#### Conclusions Based on the Sensitivity Analyses

Most importantly, we found that most of the subtle between-group thyroid hormone differences persisted when CFS patients and controls with more signs of (metabolic) low-grade inflammation were excluded, except for the occurrence of lower FT3 in CFS patients. However, FT3 below the reference range remained more frequent in CFS patients after applying stricter exclusion criteria.

#### Correlation Analysis

We found that FT3, TT3, TT4 and rT3 were positively related to hsCRP in the CFS patients (*n* = 98) and the combined controls and CFS patients (*n* = 197) (Figure S1 in Supplementary Material). TT3 and TT4 were also positively related to hsCRP in the controls (*n* = 99) (Table S3 in Supplementary Material). TSH and FT4 did not correlate with hsCRP. When combined, controls and CFS patients with low FT3 (<4.4 pmol/L) were also found to more often exhibit low hsCRP (<1 mg/L) in the whole group (*p* = 0.001) (*n* = 197; OR 6.22; 95% CI = 1.78–21.70) and in selected group 2 (*p* = 0.011) (*n* = 159; OR 4.26; 95% CI = 1.20–15.03).

## Discussion

The most remarkable observation in this case–control study was that, as a group, the present CFS patients exhibited lower FT3, TT4, TT3, %TT3, SPINA-GD, SPINA-GT, T3/T4 ratios, lower protein binding of thyroid hormones, and 24-h urinary iodine excretion, together with higher %rT3. Sixteen (16%) CFS patients exhibited the “low T3 syndrome” as compared to seven (7%) controls (Table 1). These observations were basically unaltered upon applying more stringent cutoff values for hCRP, BMI and WBC in our “sensitivity analyses” (Table S2 in Supplementary Material). CFS patients also showed some signs of (metabolic) low-grade inflammation, notably higher TC/HDL-C and ferritin, and lower HDL-C, tryptophan and kynurenine. When the TC/HDL-C ratio, HDL-C, and ferritin were evaluated according to gender, we did not find differences in the TC/HDL-C ratio in the relatively small number of men (*n* = 21), but those in the females (*n* = 77) persisted. HDL-C was lower and ferritin remained higher in both male and female CFS patients compared to controls. Therefore, we conclude that, in the present study, we found subtle evidence of low-grade (metabolic) inflammation in CFS patients. Plasma 25(OH)D below the optimal cutoff value of 80 nmol/L was found in 59% of the CFS patients and 83% of controls. Both CFS patients and controls exhibited low fish intakes, as reflected by their low omega-3 index of about 4.1 g%. An omega-3 index of 8 g% is considered to confer optimal protection against cardiovascular (62) and neuropsychiatric diseases (63).

### Comparison With NTIS

The “low T3 syndrome” encountered in a subgroup of CFS patients bears clinical chemical similarity with NTIS features. Both syndromes are biochemically characterized by low TT3 and FT3 together with normal/high-normal FT4 and normal TSH, at least in the mild and moderate forms of NTIS (36). The clinical disparity relates to the underlying severity of the diseases that are usually linked to NTIS, as opposed to the chronicity and less life-threatening nature of CFS (66). NTIS is a typical feature of critically ill patients in intensive care units, although similar changes in the HPT-axis have been observed during calorie restriction and in patients with non-critical chronic diseases, such as heart failure, chronic obstructive pulmonary disease, and diabetes mellitus (67), also referred to as mild, or atypical forms of NTIS (36, 67). All of these conditions, especially calorie restriction, might find a common denominator in an adaptive response aiming at saving energy and body protein in order to outstay any potential acute stress stimulus (68–70). Through coordinated changes in thyroid hormone metabolism, transport and receptors, NTIS might mechanistically reflect a cytokine-orchestrated allostatic condition that is remote from the well-known homeostatic negative feedback regulation of the HPT axis (71).

### Low T3 Syndrome Might Be in Line With Recent Data of the CFS Epigenome and Metabolome

A recent study identified 12,608 differentially methylated sites in peripheral blood mononuclear cells of 49 female CFS patients vs. 25 healthy female controls. These sites were predominantly involved in metabolism and to a lesser extent in neuronal cell development. Among these sites, 1,600 were related to a score of self-reported quality of life, while 13 sites were associated with glucocorticoid sensitivity in a subgroup of CFS patients with glucocorticoid hypersensitivity (72). In line with downregulated energy expenditure, recent CFS case–control studies of the metabolome revealed abnormalities in several metabolic pathways, including those reflecting mitochondrial metabolism, consistent with a hypometabolic state (73, 74). The lower proxy for DNL encountered in the currently studied CFS patients might fit into this picture, since hypothyroidism in mice has been shown to downregulate the rate limiting enzymes involved in DNL (75). In addition, induced hypothyroidism in humans for two weeks causes profound changes in FA metabolism (76). Another recent case–control study using metabolic profiling showed an altered serum amino acid profile in CFS patients, suggesting impaired mitochondrial pyruvate oxidation (74), a condition likely to reflect energy deficiency and excessive lactate production, with utilization of amino acids from endogenous protein as alternative TCA cycle substrate. The “low T3 syndrome” in a subgroup of CFS patients found in this study might be cause and consequence of the above noted epigenetic changes (72) and a driving force of the metabolic differences noted by others (73, 74) and by us. Through both genomic and non-genomic actions, T3 has profound impacts on mitochondria and metabolism (77), including several pathways regulating the expression of target genes contributing to mitochondrial biogenesis (78).

### Correlation of Thyroid Hormones With hsCRP

The association between low T3 and low hsCRP suggests that both CFS patients and controls with low FT3 are less responsive to inflammatory stimuli, which is in line with observations by others. In apparently healthy individuals, Hodkinson et al. (79) found, amongst others, that TT3 concentrations are positively related to monocyte phagocytic activity and expression of -6 (IL-6) by activated monocytes, while TT4 is positively related to CRP. Their data suggest that higher thyroid hormone concentrations within the normal range enhance innate and adaptive immunity by greater responsiveness to immune stimuli. Accordingly, Rozing et al. (80) showed that, although higher circulating levels of inflammatory markers were associated with lower levels of free serum FT3; higher serum FT3, but not higher TSH and FT4, are related to a higher production capacity of proinflammatory cytokines (IL-1β, IL-6, TNF-α) in whole blood of 85-year-old women and men, following *ex vivo* stimulation with LPS. They suggest a mutual association between T3 and proinflammatory cytokines, whereas T3 stimulates production of proinflammatory cytokines that subsequently diminish the conversion of T4 to T3. Finally, evidence of a diminished specific immune response has been found in patients with CFS. Investigating pokeweed mitogen-stimulated isolated peripheral blood mononuclear cells, Loebel et al. (81) found a deficient EBV-specific immune response in patients with CFS, possibly causing impaired EBV control. Taken together, it is possible that a subgroup of CFS patients with low FT3, but also controls with low T3, present a diminished responsiveness to immunologic stimuli.

### Comparison With Hypothyroid Patients Treated With T4 Monotherapy

Hypothyroidism is, among others, associated with a decrease in both metabolic and heart rates, oxygen consumption, body temperature and oxidation of glucose, FA, and amino acids. It has been estimated that 4–8% of genes are regulated by T3 in human skeletal muscle, rodent liver and a pituitary cell line (78). The encountered “low T3 syndrome” in our study resembles the thyroid hormone profile of a subgroup of hypothyroid patients receiving T4 monotherapy. Substitution with T4 is the currently recommended treatment of hypothyroid patients, like those with Hashimoto thyroiditis. Nevertheless, it is becoming increasingly clear that a subgroup of these patients experiences residual hypothyroid symptoms, including psychological and metabolic traces. These symptoms occur despite reaching a chemical euthyroid state, i.e., normal TSH (82, 83). In thyroidectomized rats, no single dose of T4 was able to simultaneously restore TSH, T4, and T3 in plasma and organs to normal levels (84). In so-called “euthyroid, yet symptomatic” patients, the basal metabolic rate and serum cholesterol, among others, are not fully restored and they are also likely to have both low TT3 and FT3. These findings of low T3 may be explained by a disrupted TSH-T3 shunt (41). The question whether they would benefit more from a T4 and T3 combination therapy or sustained-release T3 (85) is debated and subject of further research (82, 83). Hormone replacement therapy, notably T3, has also been suggested for severe NTIS (71, 86, 87).

In the NHANES cohort, 469 out of 9,981 participants with normal TSH were T4-treated. This subgroup of T4-treated subjects exhibited 10–15% higher TT4 and FT4, but 5–10% lower TT3 and FT3 and a 15–20% lower T3/T4 ratio, as compared to 469 carefully matched healthy controls (88). These apparently moderate differences suggest that the extra-thyroidal conversion of T4 to T3 during T4 monotherapy might be insufficient in some patients to restore serum T3 levels up to those normally maintained by an intact thyroid secreting 80% T4 and 20% T3 (82, 83, 88). A similar shift in the thyroid hormone profile was observed in the present study. However, the encountered deviations from thyroid hormone reference ranges and from controls are modest (Figure 2). It should in this context be noted that many biological effects of T3 depend on its cellular concentrations, which exhibit a complex relationship with the serum T3 concentration (89). A recent study with chemically induced hypothyroidism in rats showed a more severely reduced tissue T3 than serum FT3, averaging 1–6% of the baseline versus 30%, respectively. In addition, the extent of tissue T3 reduction, expressed as percentage of the baseline, was not homogeneous, with more serious reductions occurring in the order: liver = kidney > brain > heart > adipose tissue (90). In other words, the finding of slightly decreased circulating FT3 and perhaps also FT3 levels in the lower reference range may reflect the tip of the iceberg of the genuine T3 deficits in target tissues.

### Relation With Potential Cause(s) of CFS

Some features of CFS resemble those of a persistent response to environmental stress known as dauer (hypometabolic state). The cell danger response (CDR) is an evolutionarily conserved metabolic response, activated when a cell encounters a chemical, physical, or microbial threat that could injure or kill the cell (91). When the CDR is abnormally maintained, whole body metabolism and the gut microbiome become disturbed, the functionality of systems and organs becomes impaired and behavior is changed, resulting in chronic disease (91). Accordingly, the intestinal microbiota and virome have recently been implicated in CFS (92), while gene expression data show prominent roles for genes involved in immunity and defense (93). Psychological trauma, particularly during childhood, can also activate the CDR and produce chronic inflammation (91, 94). It has recently been shown that CFS patients are endowed with different psychological vulnerability factors, notably perfectionism and high moral standards (95). These may render them more susceptible to the psychological stress of current society, with possible effects on the immune system and thyroid axis (56, 62, 79, 80). Finally, the aforementioned case–control study by Naviaux et al. (73) showed that CFS patients present cellular metabolic responses similar to the evolutionarily conserved persistent response to environmental stress. Thus, the features of hypometabolism that characterize CFS may be a consequence of a persisting CDR, either or not inflammatory driven.

The current opinion on the causes of CFS may fit mechanistically into the presently encountered “low T3 syndrome.” We observed a shift from T3 toward rT3 in the investigated CFS patients, who exhibited lower T3/T4 ratios and higher rT3/TT3 ratios (Table 1) compared to controls. This shift toward rT3 in CFS patients was also apparent from their higher %rT3 and lower %TT3, when the sum of rT3, TT3 plus TT4 was adjusted to 100% (Table 1). These findings, as well as lower urinary iodine in CFS, may be in line with higher D3 activity. Low T3 levels in human organs have also been found in NTIS (87), but they are more likely to derive from deviant pathways of intracellular deiodination than from a seriously impaired entry of T3 into cells (87). Induction of D3 in muscle may occur in chronic inflammation (34), but D3 may also become induced by other factors, such as estradiol, progesterone, and growth hormone (96). Such mechanisms may be at the basis of CFS symptoms and may explain the lower urinary iodine excretion in CFS patients as compared with controls, although the latter also exhibited a relatively high prevalence of low iodine excretion (Table 1). Intracellular D3-catalyzed liberation of iodide from T4 and T3 may serve various antioxidant and defense functions that may potentially contribute to high intracellular “thyroid hormone consumption,” manifesting as the “low T3 syndrome” with negative iodine balance in the long term (67, 83, 97).

The lower SPINA-GD (step-up deiodinase activity) and SPINA-GT (thyroid secretory capacity) are likely to reflect thyroid allostasis responses, and the lower protein binding of thyroid hormones, as shown by the lower TT3/FT3 and TT4/FT4 ratios, may potentially result in higher metabolism/degradation of thyroid hormones. Thyroid allostasis-altered responses have been found in NTIS associated with cardiac disease (37), radiation enteritis (60) and enterocutaneous fistulas (98). The acute adaptation of thyroid hormone metabolism to critical illness may prove beneficial to the organism, whereas the more complex alterations associated with chronic illness frequently lead to type 1 thyroid allostasis (where energy demands exceed the sum of energy intake and energy mobilized from stores) (41).

### Strengths and Limitations

Strength of the present case–control study is the performing of two sensitivity analyses to assess the robustness of the association of CFS with thyroid parameters and chronic (low-grade) metabolic inflammation. These analyses resulted in some differences, but the findings in thyroid parameters remained unchanged. We also calculated the %rT3, which may be a more sensitive marker for subtle metabolic shifts than concentrations and ratios *per se*.

Our study also has limitations. There was a lack of information on the duration of illness and patient characteristics at diagnosis. For instance, dependent on illness duration, different cytokine profiles in CFS patients have been reported (99). CFS is likely a heterogeneous disease with a common final pathophysiological pathway. The present findings are possibly in line with a common final pathway, but do not get us closer to the cause(s).

## Conclusion

The most remarkable finding in this CFS case–control study was a higher prevalence of the “low T3 syndrome,” attributable to a subgroup of CFS patients. Chronic low-grade metabolic inflammation was not convincingly noted. Low circulating T3 may reflect more severely depressed tissue T3 levels. The “low T3 syndrome” might be in line with recent metabolomic studies pointing at a hypometabolic state. It also resembles a mild form of NTIS and the low T3 syndrome experienced by a subgroup of hypothyroid patients with T4 monotherapy. Our study needs confirmation and extension by others. If confirmed, trials with, e.g., T3 and iodide supplements might be indicated.

## Ethics Statement

All patients and controls received a verbal and written explanation of the objectives and procedures and all provided us with written informed consent. The study was in agreement with the ethical standards of the responsible committee on human experimentation (institutional and national) and with the Helsinki Declaration of 1975, as revised in 2008. The protocol was approved by the University Medical Center Groningen (UMCG) Medical Ethical Committee (NL44299.042.13, METc 2013/154, dated August 12, 2013).

## Author Contributions

BR-N, DD-B, and FM designed the research; BR-N and RT conducted the research; BR-N, RT, and EV analyzed the samples and data; BR-N, DD-B, and FM wrote the article; and FM had primary responsibility for final content. All authors read and approved the final manuscript.

## Conflict of Interest Statement

The authors declare that the research was conducted in the absence of any commercial or financial relationships that could be construed as a potential conflict of interest.

## Abbreviations

AA, arachidonic acid; AMC, Academic Medical Center; CDR, cell danger response; CFS, chronic fatigue syndrome; CI, confidence interval; D3, deiodinase 3; DHA, docosahexaenoic acid; DNL, *de novo* lipogenesis; ELN, European Laboratory of Nutrients; EPA, eicosapentaenoic acid; FA, fatty acids; FT3, free triiodothyronine; FT4, free thyroxine; GD, sum activity of deiodinases; GT, secretory capacity of the thyroid gland; Hb, hemoglobin; HDL-C, High Density Lipoprotein-cholesterol; HPA, hypothalamus–pituitary–adrenal; HPT, hypothalamus–pituitary–thyroid; hsCRP, high-sensitive C-reactive protein; IL-1, interleukin-1; IL-6, interleukin-6; LPS, lipopolysaccharides; TNF, tumor necrosis factor; NFκB, nuclear factor kappa B; NTIS, non-thyroidal illness syndrome; RBC, red blood cells; rT3, reverse T3; SPINA, structure parameter inference approach; sTSHi, standard TSH index; T2, 3,3′-diiodothyronine; TC, total cholesterol; TSH, thyrotropin; TT3, total triiodothyronine; TT4, total thyroxine; UMCG, University Medical Center Groningen; WBC, white blood cells.
