# Supplementary material for: Higher Prevalence of “Low T3 Syndrome” in Patients With Chronic Fatigue Syndrome: A Case–Control Study
Source: Front Endocrinol (Lausanne). 2018 Mar 20;9:97. doi: 10.3389/fendo.2018.00097 (PMC5869352; doi:10.3389/fendo.2018.00097)
Supplement: Supplementary file 4 [file image_1.PDF]

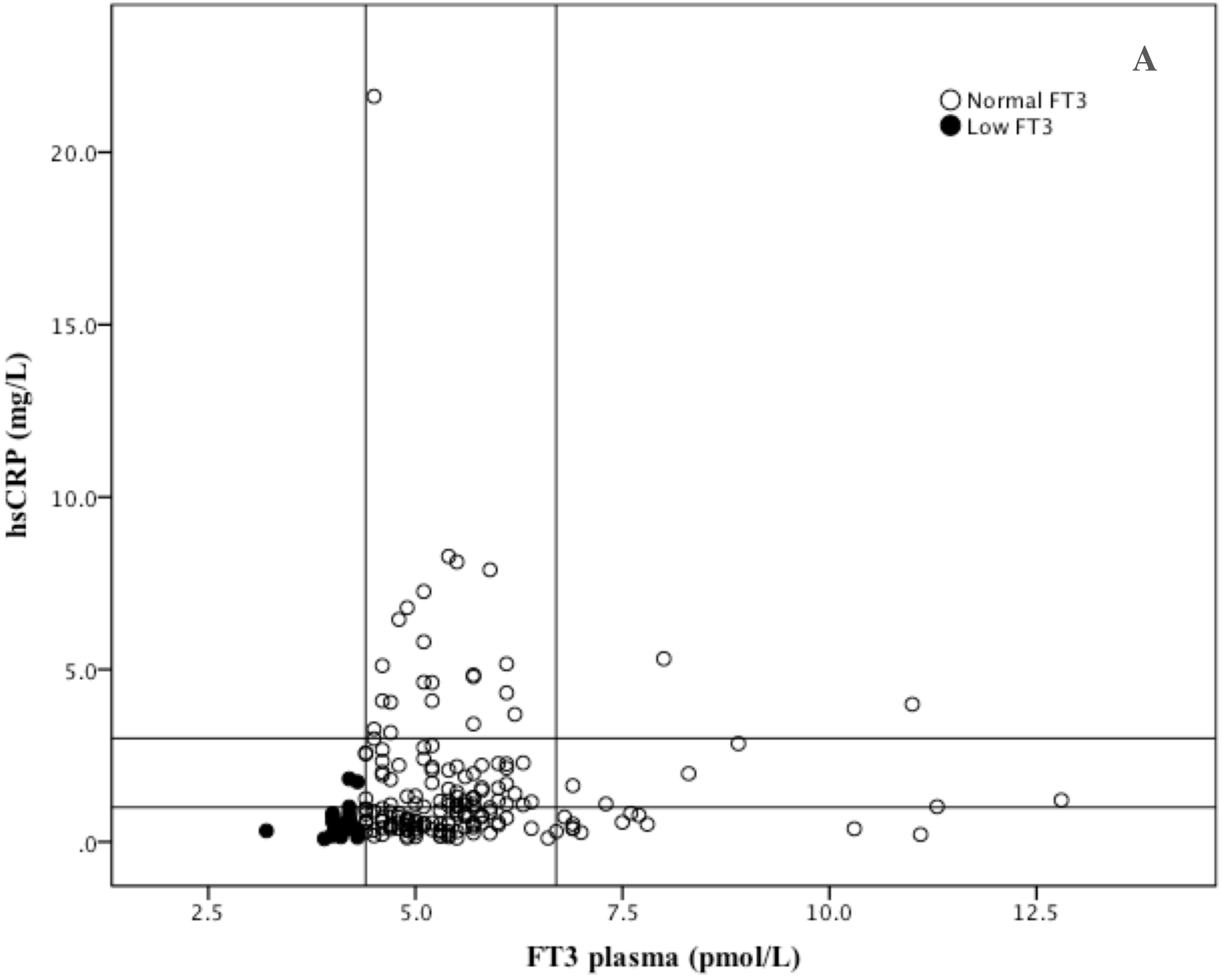
A
Normal FT3
Low FT3
hsCRP (mg/L)
20.0
15.0
10.0
5.0
.0
2.5
5.0
7.5
10.0
12.5
FT3 plasma (pmol/L)

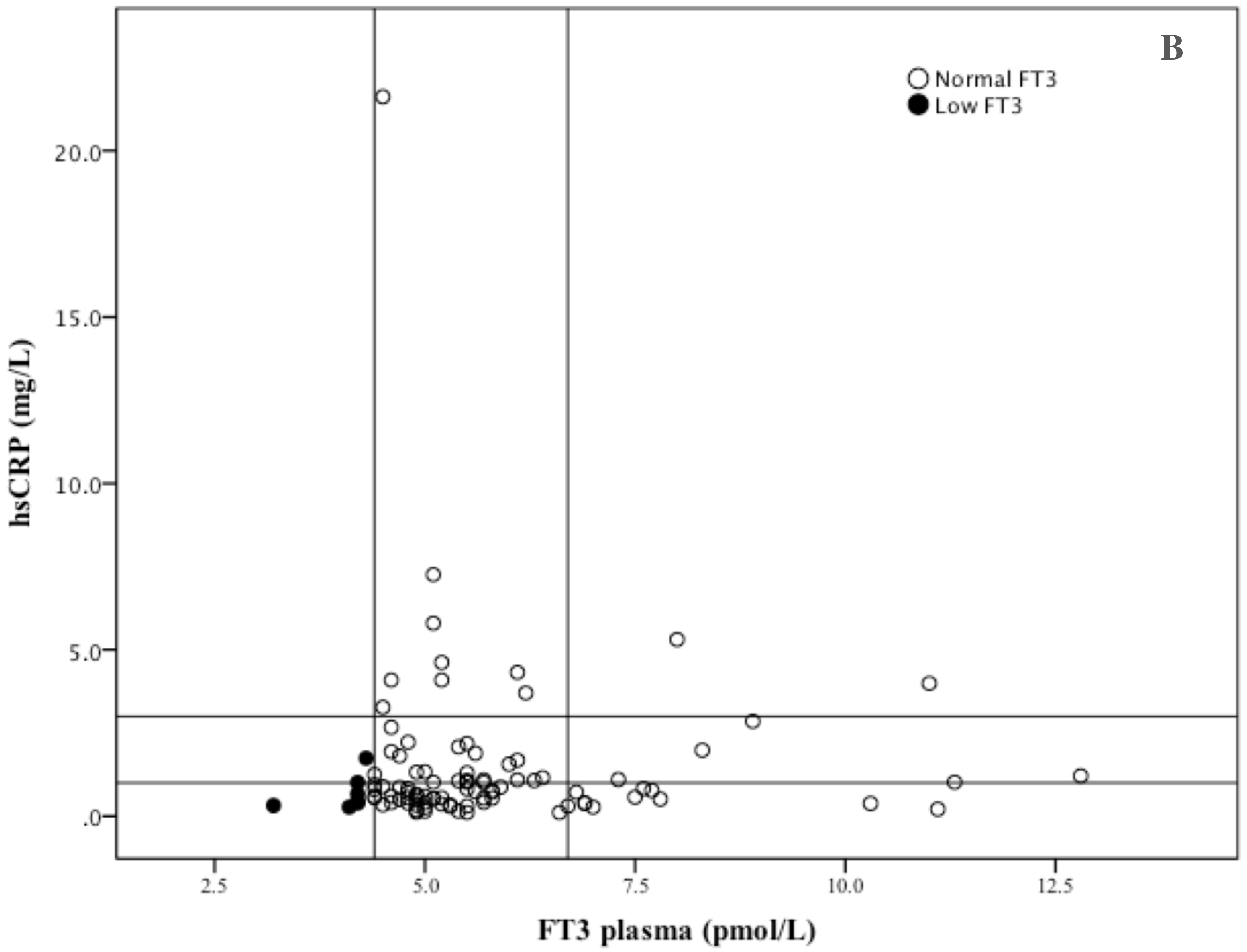
B
Normal FT3
Low FT3
hsCRP (mg/L)
20.0
15.0
10.0
5.0
.0
2.5
5.0
7.5
10.0
12.5
FT3 plasma (pmol/L)

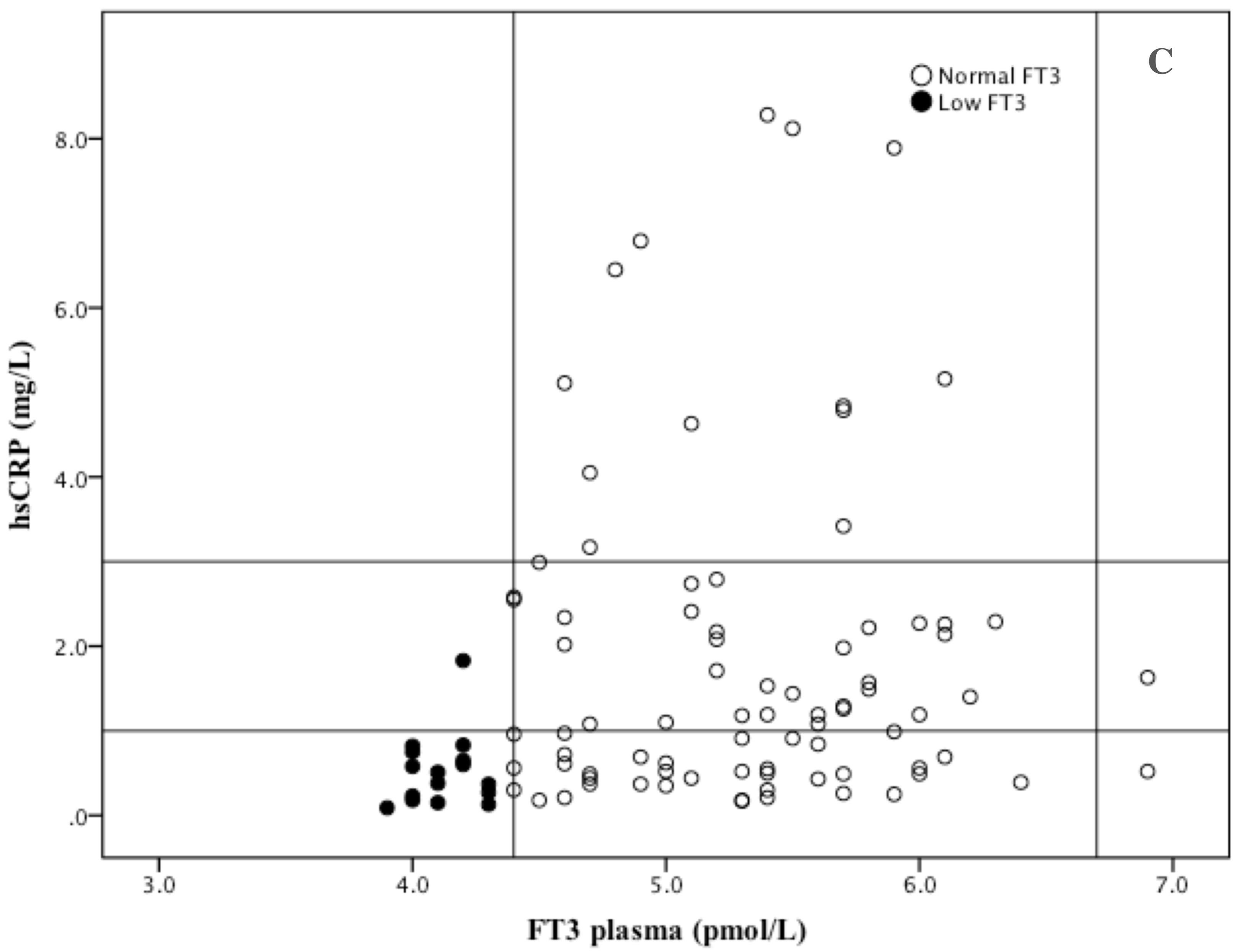

C
Normal FT3
Low FT3
hsCRP (mg/L)
8.0
6.0
4.0
2.0
.0
3.0
4.0
5.0
6.0
7.0
FT3 plasma (pmol/L)

Supplemental Figure 1. Relationships between FT3 and hsCRP in the whole group, controls and CFS patients.

Panel A represents the whole study group (both patients and controls); panel B, controls; and panel C, CFS patients. Vertical lines represent FT3 reference values. Horizontal lines represent hsCRP=3 mg/L and 1 mg/L.

Abbreviations: FT3, free T3; hsCRP, high-sensitive C-reactive protein.
